# A Quantitative and Standardized Method for the Evaluation of Choroidal Neovascularization Using MICRON III Fluorescein Angiograms in Rats

**DOI:** 10.1371/journal.pone.0128418

**Published:** 2015-05-29

**Authors:** Jonathan P. Wigg, Hong Zhang, Dong Yang

**Affiliations:** 1 Centre for Eye Research Australia, University of Melbourne, Royal Victorian Eye and Ear Hospital, East Melbourne, Victoria, Australia; 2 Eye Hospital, Harbin Medical University, Nangang District, Harbin, Heilongjiang Province, China; Medical University of South Carolina, UNITED STATES

## Abstract

**Introduction:**

In-vivo imaging of choroidal neovascularization (CNV) has been increasingly recognized as a valuable tool in the investigation of age-related macular degeneration (AMD) in both clinical and basic research applications. Arguably the most widely utilised model replicating AMD is laser generated CNV by rupture of Bruch’s membrane in rodents. Heretofore CNV evaluation via in-vivo imaging techniques has been hamstrung by a lack of appropriate rodent fundus camera and a non-standardised analysis method. The aim of this study was to establish a simple, quantifiable method of fluorescein fundus angiogram (FFA) image analysis for CNV lesions.

**Methods:**

Laser was applied to 32 Brown Norway Rats; FFA images were taken using a rodent specific fundus camera (Micron III, Phoenix Laboratories) over 3 weeks and compared to conventional ex-vivo CNV assessment. FFA images acquired with fluorescein administered by intraperitoneal injection and intravenous injection were compared and shown to greatly influence lesion properties. Utilising commonly used software packages, FFA images were assessed for CNV and chorioretinal burns lesion area by manually outlining the maximum border of each lesion and normalising against the optic nerve head. Net fluorescence above background and derived value of area corrected lesion intensity were calculated.

**Results:**

CNV lesions of rats treated with anti-VEGF antibody were significantly smaller in normalised lesion area (p<0.001) and fluorescent intensity (p<0.001) than the PBS treated control two weeks post laser. The calculated area corrected lesion intensity was significantly smaller (p<0.001) in anti-VEGF treated animals at 2 and 3 weeks post laser. The results obtained using FFA correlated with, and were confirmed by conventional lesion area measurements from isolectin stained choroidal flatmounts, where lesions of anti-VEGF treated rats were significantly smaller at 2 weeks (p = 0.049) and 3 weeks (p<0.001) post laser.

**Conclusion:**

The presented method of in-vivo FFA quantification of CNV, including acquisition variable corrections, using the Micron III system and common use software establishes a reliable method for detecting and quantifying CNV enabling longitudinal studies and represents an important alternative to conventional CNV quantification methods.

## Introduction

Age-related Macular Degenerating (AMD) is the leading cause of vision loss in Australia, Europe, and the United States [1]. The prevalence of AMD is expected to double in the coming decades as industrialised countries population increase in age [2]. Choroidal neovascularization (CNV), the development of neovascularization extending from the choroid into the sub-retinal space, is the main cause of severe vision loss in AMD [3].

Retinal research, including the investigation of novel therapies for CNV, relies heavily on the use of animal models. Small rodents constitute the majority of disease models that have been developed to date [4]. Therefore, assessment of the rodent retina has become increasingly important in basic and preclinical research and furthers the understanding of human retinal disease. Currently the standard animal model of CNV used for treatment-evaluation experiments is laser induced rupture of the Bruch’s membrane in mice and rats. This model is a well-established [5–8] derivative of work done with primate CNV research[9] and is a convenient, inexpensive and reliable way of replicating pathogenic choroidal neovascularization. The model replicates the complex processes involved in exudative AMD reliably, particularly with regards to pathological angiogenesis, including the release of potent angiogenic factors, and the subsequent inflammatory response [4].

Heretofore quantitative evaluation of disease progression has been accomplished with tissue specimens obtained after euthanasia using routine choroidal flat mount preparation with 2D [10–12] or 3D [10] image software analysis of CNV area at predetermined endpoints. Even though this technique represents the current gold standard for neovascular quantification, it is quite technically demanding, time consuming and image quantification is subject to variation by choice of analytical software[13]. Fundus Fluorescein Angiography (FFA) offers many advantages over choroidal flat mounting as it allows high-throughput, non-invasive in vivo assessment of CNV that may be performed longitudinally in the same animal, reducing inter-animal variability and, potentially, the number of animals required in some experiments. Additionally FFA allows vessel permeability assessment as well as standard CNV area measurement. FFA while common place in clinical applications including diagnosis and tracking disease progression, has failed to be incorporated into basic research due to technical limitations in small animals and lack of quantifiable method of analysis. Due to small size, high optical power, large refractive error and strong negative spherical aberration in rodents [14,15], attempts to use fundus cameras designed for larger mammals have produced limited results [16–19]. Moreover, analysis of rodent FFA images has been largely qualitative, using subjective grading systems varying from study to study [16,20,21].

The Micron III (Phoenix Laboratories) fundus camera has been developed to produce high resolution colour fundus images as well as FFA suitable for CNV assessment specifically for rodents [22–25] with a transverse resolution of 8μm for rats and <4μm for mice. Despite these pioneering advances in this field, as far as we know, the standardized use of the Micron III to conveniently quantify CNV has not been reported. Herein, we aim to optimize the technique to quantify CNV in a standardized, consistent, and reproducible way as well as explore factors influencing FFA imaging using the Micron III fundus imaging system in rats and its impact on data obtained. The standardized approach was subsequently used to characterize avascular laser burns and CNV lesions in rats receiving either intravitreal PBS acting as a negative control or a well-established inhibition of angiogenesis by anti-VEGF antibody [21]. The presented data provides evidence that our method allows for the quantitative analysis of both laser spots and treated and untreated CNV using FFA. Using our standardised approach, FFA becomes a beneficial alternative to post-mortem measurements using routine flat mount histology allowing quantifiable long term longitudinal tracking of CNV progression in vivo, assisting the development of novel therapies to severe sight impairing disorders such as AMD and Diabetic Retinopathy.

## Methods

### Ethics Statement

Animal experiments were conducted with the approval of the Royal Victorian Eye and Ear Hospital (RVEEH) Animal Research & Ethics Committee (AREC) and were consistent with the Association for Research in Vision and Ophthalmology statement (ARVO) for the Use of Animals in Ophthalmic and Vision Research.

### Animals and Laser induced CNV and laser- treatment without CNV generation

Adult Brown Norway rats weighing 297±19 grams (mean±SD) each were maintained in a 12-hour light/12-hour dark cycle with a room illuminance of 140 to 260 lux during the bright portion of the cycle. Animals were provided standard food and water ad libitum. In vivo experiments were conducted under general anaesthesia by intraperitoneal injection of ketamine 75mg/kg, xylazine 10mg/kg. Care was taken to use rats of the same approximate age(10 weeks ± 1 week) in control and experimental groups [26], and female and male animals were evenly distributed between the treatment groups [27].

Laser treatment was performed as previously described [21] in all animals. In brief, after general anaesthesia, the pupils were dilated using tropicamide (5mg/mL). The animals were then positioned in front of an argon laser and a coverslip with Hypromellose coupling gel applied to the eye. Settings for laser coagulation were 100μm spot size, 100ms emission time and 150mW laser-energy at 532 nm emission wavelength. Using a slit lamp, 3–5 laser burns per eye were applied radially 2–3 disc diameters from the optic nerve with development of a small white bubble as confirmation of Bruch’s membrane rupture for CNV formation. Absence of bubble formation indicated chorio-retinal burn alone without CNV development, and will henceforth be referred to as ‘CR Burn’. Approximately 75% of applied laser was successful in generating CNV. Rats exhibiting vitreal haemorrhage and obscuring the posterior segment were excluded from fundus imaging analysis.

To ascertain differences in fluorescein administration route on FFA image quality, 4 rats were given laser treatment and FFA performed with either intraperitoneal or intravenous fluorescein injection.

All remaining rats received laser treatment resulting in CNV generation in the left eye as described and were randomly allocated into 2 groups. Group 1 (n = 16), received intravitreal injection of 5μL anti-VEGF polyclonal antibody (AF564, R&D Systems; 25μg/mL) in the left eye immediately post laser treatment and 7 days post laser. Group 2 (n = 16), acting as a negative control, received a sham intravitreal injection of the same amount of sterile PBS into the left eye and readministered 7 days later. FFA imaging, choroidal flat mount and histology were performed at scheduled times.

### Micron III Image Acquisition

Rats were generally anesthetized by intraperitoneal injection, per laser procedure, and placed on a heating pad before *in vivo* imaging. Pupils were dilated with tropicamide (5 mg/mL) drops. Dilation of the lateral tail vein was achieved by placing the animal under a heat lamp. The animals were positioned on the Micron III stage and Hypromellose coupling fluid applied to the eye. The camera and eye position was adjusted ensuring correct alignment and focus on the optic nerve head plane using standard colour fundus photography before adjusting to the appropriate filter set for Fluorescein Angiography. 0.1 mL/kg, 10% fluorescein sodium was then administered via intraperitoneal injection or intravenous injection through the lateral tail vein. The fluorescein bolus was delivered over 3 seconds; simultaneously with the first image capture. Images were captured using the Streampix Software (Phoenix Research Laboratories) as XGA resolution (1024x768 pixels) 24bit RGB sequential tiff files with the Micron III light source at maximum intensity and gain setting of +4 db. Images were taken at 7 day intervals for 3 weeks after laser treatment. FFA images with fluorescein administration by intraperitoneal injection were taken 1 frame/second for 120 seconds, and 1 frame/5 seconds thereafter up to 10 minutes post injection. For intravenously administered fluorescein, images were taken at 30 frames per second for 120 seconds. All FFA image analysis was performed with open-source software, ImageJ (National Institute of Mental Health, Bethesda).

### Lesion area measurement and Quantitative Intensity Level Analysis of Micron III FFA images

For area measurement, images were imported into ImageJ, where trained graders blinded to the experimental treatment, determined lesion area and intensity.

**Fig 1:**
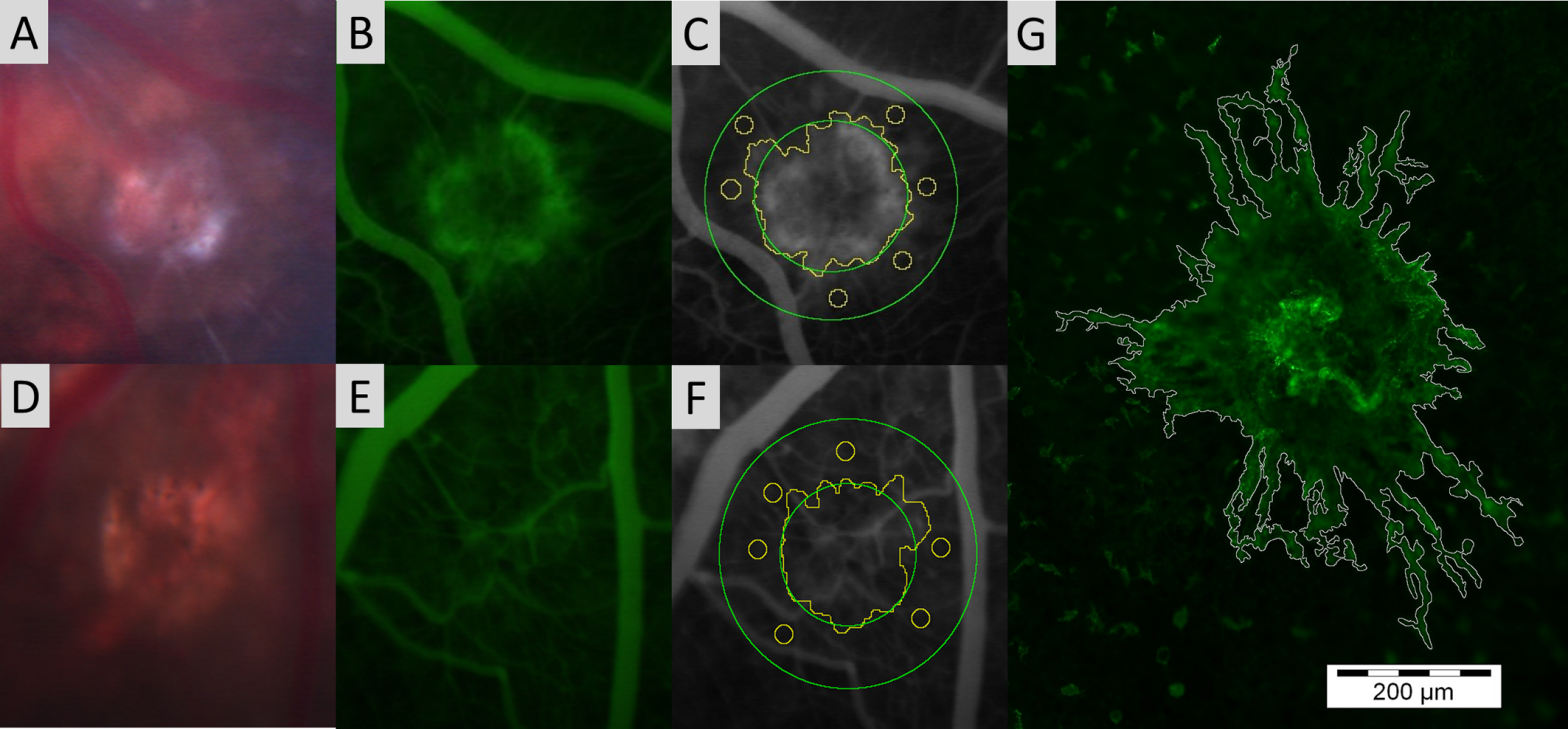
Illustration of area and intensity assessment of CNV lesions in FFA and choroidal flatmount images including corresponding colour Ffundus and unmodified FFA. (A-C): Colour fundus, FFA and representative hyperfluorescent CNV lesion analysis by freehand selection tool in ImageJ under digital magnification of the green channel from a FFA image produced by the Micron III system. Native background fluorescence intensity avoiding choroidal vessels was measured within a defined annulus (green) around the lesion. (D-F): Corresponding images of lesion analysis for chorio-retinal burn. (G): Representative CNV lesion analysis by freehand selection tool in ImageJ of Isolectin GS-IB4-AF488 stained choroidal flatmount. *Note scale bar applicable to Fig 1G only.

RGB Tiff files were imported into ImageJ and individual colour channels separated. Red and Blue Channels were removed and all area and intensity values were measured from the Green Channel. Under digital magnification (800%) and using the ‘freehand selection tool’ the maximal border of each hyperfluorescent CNV lesion or the hypofluorescent laser burn was determined and the area recorded in pixels (Fig 1). Unavoidable magnification of fundus images to varying extent is introduced by the lens system exerting pressure on the corneal surface. As such each CNV or CR burn area was normalised against the averaged optic nerve head area (determined in an identical manner to lesion area measurements). The normalised CNV area was then plotted against time after laser treatment.

The fluorescence intensity (average grey level) within the maximum border of each CNV lesion and CR burn was calculated using ImageJ, areas where large vessels overlapped the hyperfluorescent area was excluded (Fig 1). Background fluorescence intensity was measured by defining an annulus area around the CNV lesion, from which, six representative areas avoiding retinal vessels were identified and an average background fluorescence intensity value calculated. The inner border of the annulus was defined as the outer limit of the CNV lesion while the outer border of the annulus was defined using twice the radius of the inner border. The net fluorescence intensity above background was thus calculated by subtracting the calculated background value from the CNV hyperfluorescence intensity.

Net lesion fluorescence from each sequential fundus image was calculated and plotted against time after fluorescein injection via intravenous and intraperitoneal administration. The time corresponding to peak fluorescence intensity was identified and this value was used for all subsequent intensity calculations.

The average ‘area corrected CNV fluorescent intensity’ of the hyperfluorescent regions was calculated by multiplying the ‘net fluorescence intensity’ of individual lesions by the ‘normalised lesion area’; The calculated value represents the sum of the grey value within the bounds of the lesion border normalised against the optic nerve head, allowing quantitative value for the severity of CNV lesions.

### Choroidal Flat Mount Preparation

Rats were euthanized by intraperitoneal injection of phenobarbital (20%, Lethobarb), and the eyes were enucleated and fixed in 4% paraformaldehyde for 90 minutes at 4°C. Under dissecting microscope the eyes were cut through the corneal limbus and the anterior segment, including lens, removed. 4 to 5 cuts were made in the resulting eye cup perpendicular to the pars plana toward the optic nerve head, ensuring cuts were away from CNV lesions, The neuro-retina was removed using fine forceps and the sclera-choroid-retinal pigment epithelial complex was isolated and incubated in a blocking solution (0.5% BSA, 0.5% triton-X) for 4 hours at 4°C. ‘Choroidal’ flatmounts were then incubated for 16 hours at 4°C in Isolectin GS-IB4 conjugated with Alexa Fluor 488 (1:100 dilution, 500ug/ml). Flat-mounts were washed three times in PBS, mounted in Dako Fluorescent Mounting Medium and coverslipped. Fluorescent micrographs of the choroidal flatmounts were then taken using Olympus DP-71 camera system attached to an Olympus BX61 microscope.

### Hyperfluorescent area measurement of choroidal flatmount micrographs

Measurement of CNV lesions was done using two methods by masked graders. Fluorescent micrographs were imported into ImageJ, where the maximal border of the CNV lesion was manually outlined under digital magnification and the encompassed area measurement in pixels converted to μm^2^ using the scale bar (Fig 1). CNV area was also assessed in a similar fashion as described by Toma et al.[13] using Adobe Photoshop (Adobe Systems Inc., San Jose, CA). In brief, the border of the hyperfluorescent area was determined utilising the lasso tool in. Areas were calculated in pixels and converted to μm^2^ using the scale bar.

### Haematoxylin and Eosin staining

Rats were euthanized by intraperitoneal injection of phenobarbital (20%, Lethobarb), and whole eyes were enucleated and immediately fixed in Davidson’s fixative for 16 hours at room temperature. Eyes were then rinsed and stored in 70% Ethanol at 4°C until processing. Whole globes were then processed via standard overnight automated paraffin processing technique through a series of graded alcohols and histolene, followed by paraffin embedding. Paraffin blocks were then sectioned at 5 μm and floated onto 3-aminopropyltriethoxysilane (APES) coated slides. Routine haematoxylin and eosin staining was performed on selected slides before being mounted in DPX mountant (Sigma), and coverslipped.

### Statistical Analysis

Two-tailed Student’s t-tests were used to compare normalised area calculations, net fluorescent intensity measurements and fluorescent density calculations between groups unless otherwise stated. Data is reported as mean ± SD. Differences are regarded as significant when *p*<0.05.

## Results

### Fluorescein Administration Delivery Route Comparison

**Fig 2:**
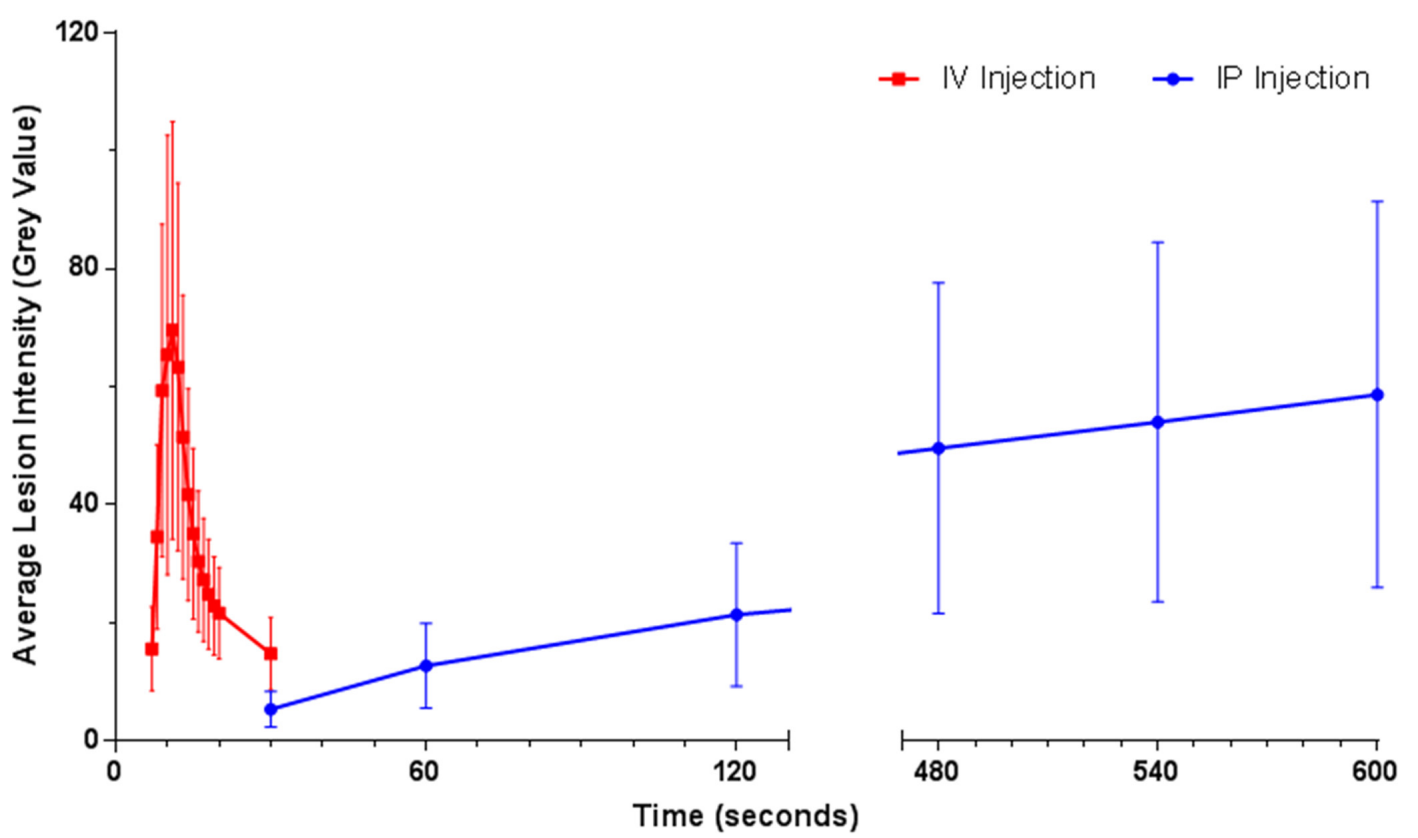
Fluorescein Administration Route CNV Intensity Comparison. CNV intensity analysis of unprocessed FFA images with fluorescein administration by intravenous (IV) and intraperitoneal (IP) injection. Each time point represents the mean grey value ± SD (n = 4). FFA images with IP fluorescein were taken at 1 frame/second for 120, and 1 frame/5 seconds thereafter up to 10 minutes post injection. IV fluorescein angiograms were taken at 30 frames/second for 120 seconds. Intensity data from 130–470 seconds not shown.

In a routine angiogram, with fluorescein introduced intravenously, the intensity of fluorescence would always be expected to reach a peak during initial dye transit and then decrease with time in our study. Intravenous fluorescein administration via the lateral tail vein resulted in rapid progression through classical phases of clinical angiography and similar phase duration was observed. We observed a fluorescent flux of approximately 15 seconds in duration with peak fluorescein intensity achieved in the retinal vasculature and choroid at 10.11±1.27 seconds, with peak CNV lesion fluorescence momentarily later at 10.22±1.30 seconds (Fig 2, Table 1). The total transition time from choroidal ‘flush’/arterial stage to late venous stage takes approximately 7–8 seconds, with choroidal ‘flush’/arterial stage initiating 6.5 seconds after injection (Table 1). Intraperitoneal administration of fluorescein delays the appearance in the retinal vasculature, while the calculated intensity values (with respect to time) mimic a logarithmic function (Fig 2). At the end of the 10-minute observation time CNV fluorescent intensity was still increasing, with the first appearance in the retinal vasculature 22.5±8.7 seconds after initial intraperitoneal administration. The rate at which CNV fluorescence intensity increased was highly variable between animals with intraperitoneal injection. On this basis, the following experiments were undertaken using intravenous fluorescein injection at 10.2 seconds post injection, correlating to peak fluorescence for the intensity quantification of both CNV and non-vascular lesions for all rats.

**Table 1:** Appearance and peak fluorescent intensity times post injection via alternate fluorescein administration routes.

	Intravenous Injection		Intraperitoneal Injection	
	Appearance time (seconds) ±SD	Peak time (seconds) ±SD	Appearance time (seconds) ±SD	Peak time (seconds) ±SD
**Optic Nerve**	7.18±1.17	10.11±1.27	22.58±8.59	600+
**Choroid**	7.27±1.10	10.11±1.27	32.83±13.36	600+
**Blood Vessels**	6.55±0.93	10.11±1.27	22.50±8.68	600+
**CNV**	7.45±0.93	10.22±1.30	33.33±13.25	600+

Note: Values represent average fluorescein appearance time/peak intensity in seconds ± standard deviation.

### CNV area measurement on fluorescein angiogram, confirmed by conventional flatmount technique and histological findings

**Fig 3:**
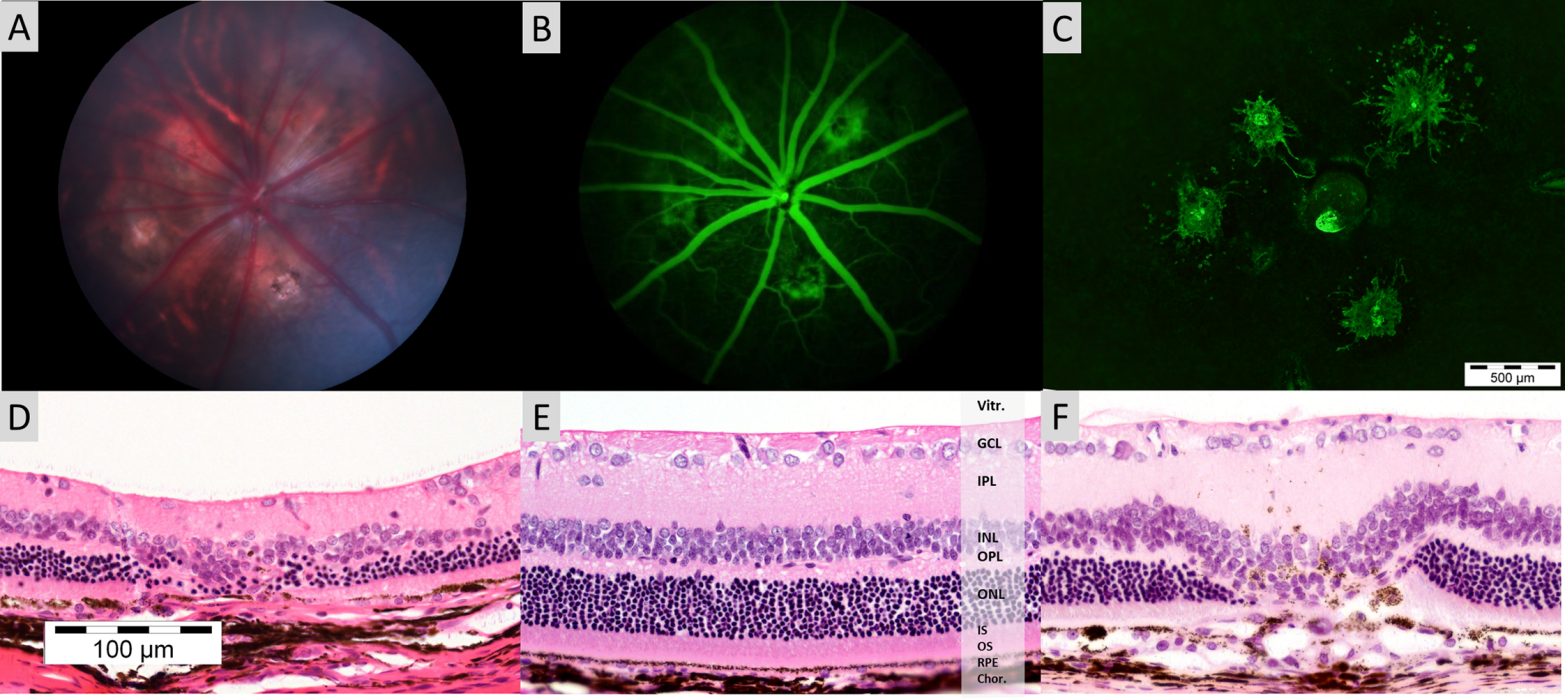
Representative Micron III Images with Micrographs of conventional Histopathological preparations. Colour fundus photo (A) and fluorescein angiogram (B) of a Brown Norway rat exhibiting 4 choroidal neovascular lesions generated by rupture of the Bruch’s membrane by laser. Fluorescein angiogram (B) taken at 10.2 seconds post intravenous injection, corresponding to peak CNV fluorescence. Corresponding choroidal flatmount image (C) of the same eye taken at 2 weeks post laser stained with Isolectin-IB4 conjugated with Alexa Fluor 488. *Scale bar represents 500μm and is applicable to Fig 3C only. Representative micrograph of haematoxylin and eosin stained section of (D) Chorio-Retinal Burn at 3 weeks post laser (E) Retina without laser treatment (F) CNV lesion at 3 weeks post laser (D) Classical fusiform shaped sub retinal neovascular lesions are observed in both treatment groups confirming CNV formation by Bruch’s Membrane rupture by laser. *Scale bar represents 100μm and is applicable to Fig 3D, 3E and 3F only. *(Vitr = Vitreous, GCL = Ganglion Cell Layer, IPL = Inner Plexiform Layer, INL = Inner Nuclear Layer, OPL = Outer Plexiform Layer, ONL = Outer Nuclear Layer, IS = Inner Segment, OS = Outer Segment, RPE = Retinal Pigment Epithelium, Chor. = Choroid)

Representative colour fundus and FFA images of CNV lesion taken with the Micron III camera system and conventional flat mount 2 weeks post laser are show in Fig 3. Increased permeability and reduced fluorescein clearance in the newly formed choroidal vessels leads to persistent hyperfluorescent regions, while ablation of choroidal vessels by laser burn without CNV generation leads to persistent hypofluorescent regions. Corresponding lesion areas were calculated from FFA images at 10.2 seconds post fluorescein injection and normalised against the area of the optic nerve head. Poor lesion border definition was observed 1 weeks post laser, as such, area and intensity calculations were not included in analysis.

**Fig 4:**
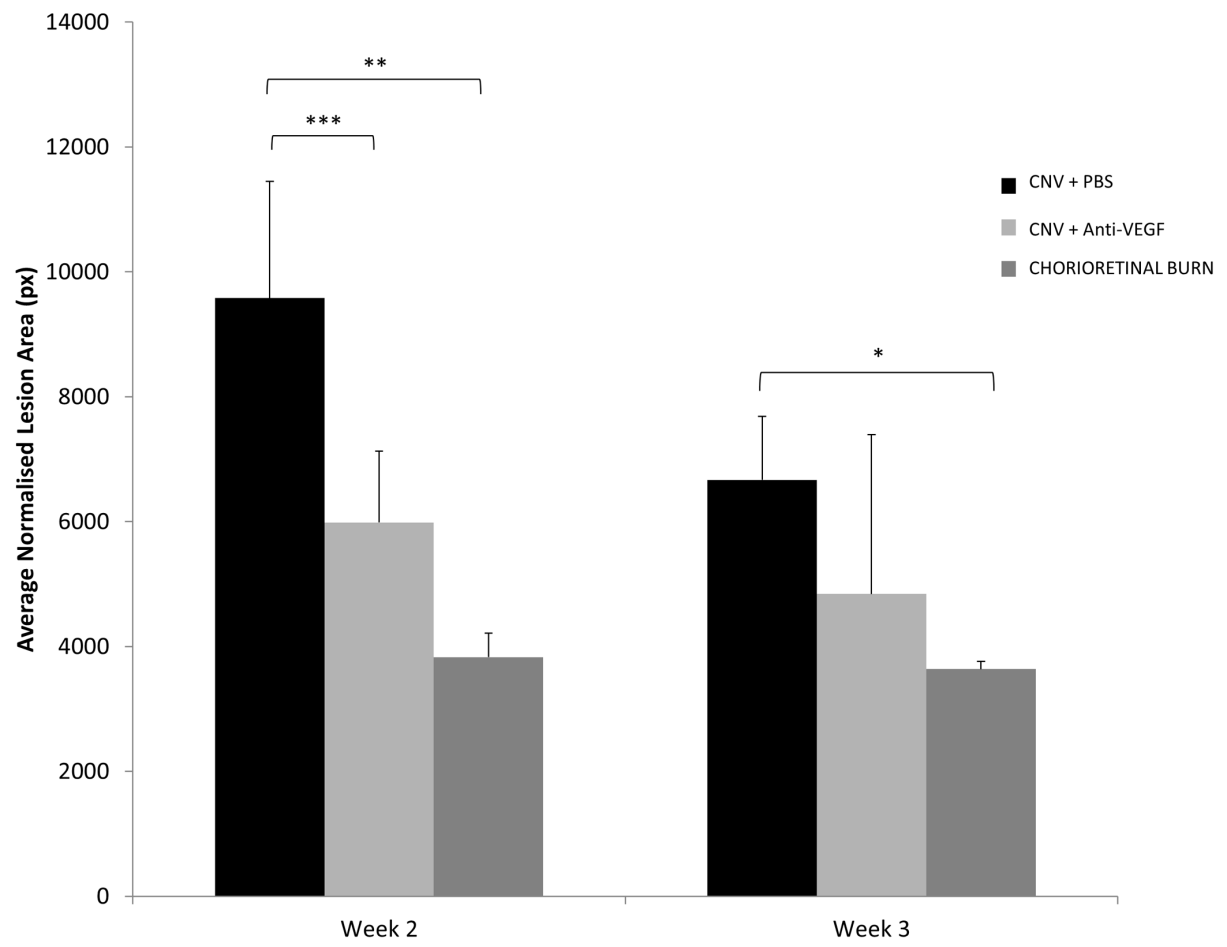
Fluorescein Angiogram CNV Area Analysis. Calculated area of Laser Burn without CNV and CNV lesions receiving anti-VEGF treatment versus PBS treatment of FFA images using manual border delineation by trained specialists blinded to experimental treatment in ImageJ, normalised to optic nerve head area. Values represent mean lesion area in pixels ± SD (n = 16). * Denotes statistically significant (*p*<0.05) difference in the calculated normalised lesion area tested by two-tailed Student’s t-test. ** Denotes statistically significant (*p*<0.01) difference in the calculated normalised lesion area tested by two-tailed Student’s t-test. *** Denotes statistically significant (*p*<0.001) difference in the calculated normalised lesion area tested by two-tailed Student’s t-test.

The calculated lesion area of the avascular CR burn was significantly (*p =* 0.0032) smaller than the PBS treated CNV at week 2, though no significant difference in area was observed between avascular laser spots and CNV lesions of anti-VEGF treated animals. Additionally, avascular CR burns remained constant in size over the observation period (Fig 4).

**Fig 5:**
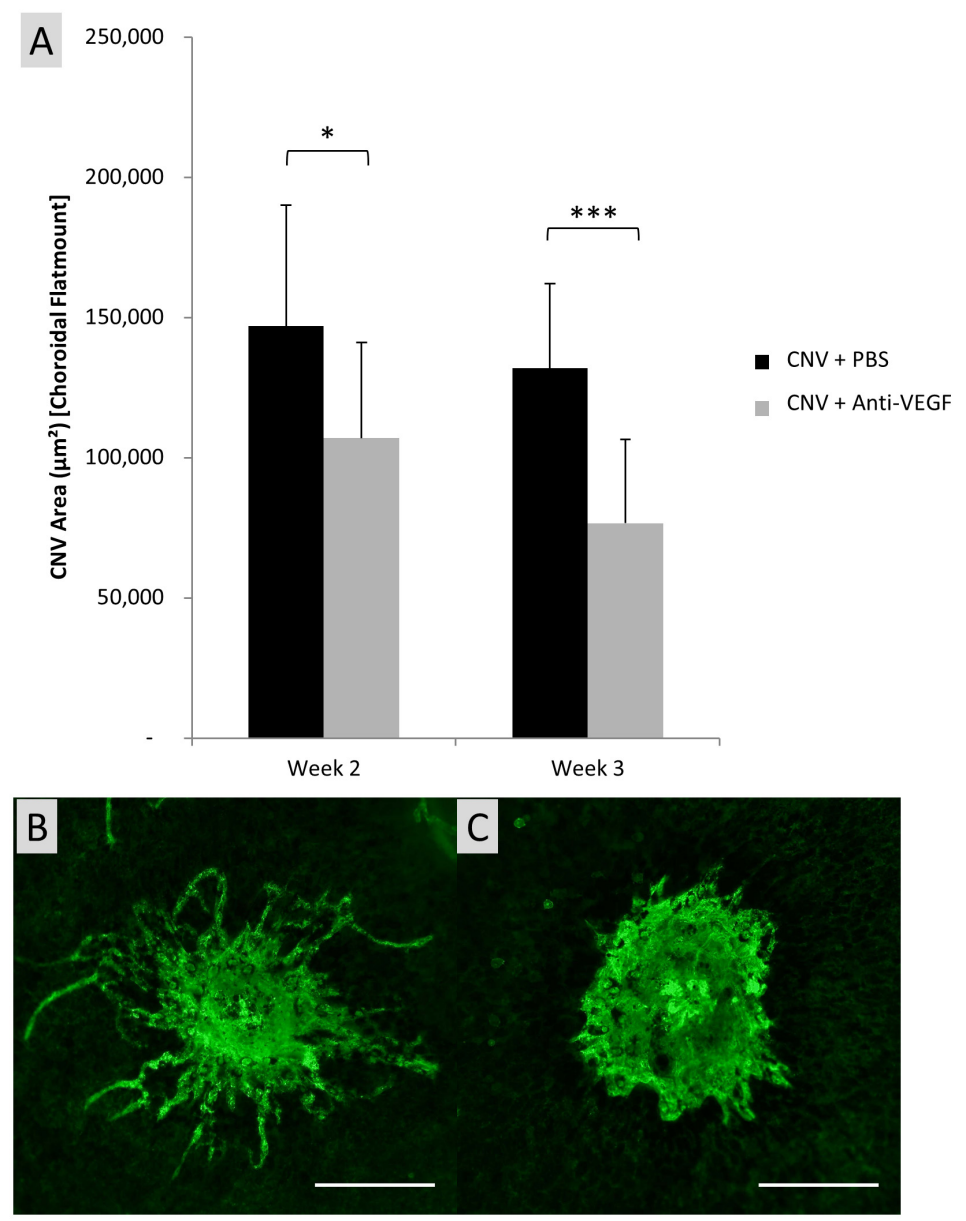
Representative micrographs of CNV lesions using choroidal flatmount and corresponding area calculation. (A) Calculated CNV area on choroidal flatmounts using free hand selection technique in ImageJ, adjusted from pixels to μm². Each column represents the mean area ± SD (n = 16). Representative fluorescent micrographs of neovascular lesions of PBS injected (B) and anti-VEGF treated eyes (C) at 2 weeks post laser, produced by routine choroidal flatmount and stained with Isolectin-IB4 conjugated with Alexa Fluor 488. Note reduced vascular budding in anti-VEGF treated eyes at 2 weeks. * Denotes statistically significant (*p*<0.05) difference in the measured CNV area tested by two-tailed Student’s t-test. *** Denotes statistically significant (*p*<0.001) difference in the measured CNV area tested by two-tailed Student’s t-test.

As anticipated, CNV of anti-VEGF treated rats was significantly smaller (p<0.001) than PBS treated animals (Fig 4) at week 2 post laser treatment. Interestingly the average area of CNV lesions at week 3 was reduced in both PBS and anti-VEGF treatment groups and anti-VEGF treated rats experienced larger than normal variation in CNV area during week 3. Conventional choroidal flatmount measurement of CNV lesions of anti-VEGF treated rats (107030±34051μm²) were shown to be significantly (*p* = 0.049) smaller than PBS treated lesions (146951±43160μm²) at week 2 (Fig 5). The central portion of the lesion in both treatment groups appears approximately comparable however a distinct lack of vascular budding is apparent in anti-VEGF treated rats.

Conventional flatmount technique correlated with area measurements obtained by FFA; however the reduction in average area measurements was more pronounced in FFA measurements. Though large, consistent variation in lesion area was observed in choroidal flatmounts across all treatment groups and time points. No significant difference in lesion area measurement was observed between Anti-VEGF treated rats and PBS treated rats at week 3, yet significant difference in lesion area was calculated between treatment groups at this time using flatmount measurements.

Confirmation of CNV generation by laser was shown in haematoxylin and eosin staining of paraffin embedded sections from PBS and anti-VEGF IgG treated eyes, a representative image is shown in Fig 3F. Rats exhibit classic large fusiform-shaped lesions of fibrovascular proliferations infiltrating the retina. Large inner-retina vessels and formation of an RPE monolayer separating the neural retina from the underlying lesion can be observed. Additionally, pigmented macrophage-like cells appear within the CNV lesion. CR burns without CNV formation are subject to the laser impact site and retention of intact Bruch’s Membrane; Fig 3D shows a representative lesion where the laser has impacted the outer retina, resulting in significant loss of the outer plexiform layer, outer nuclear layer and inner and outer segments.

### Net Fluorescent Intensity

**Fig 6:**
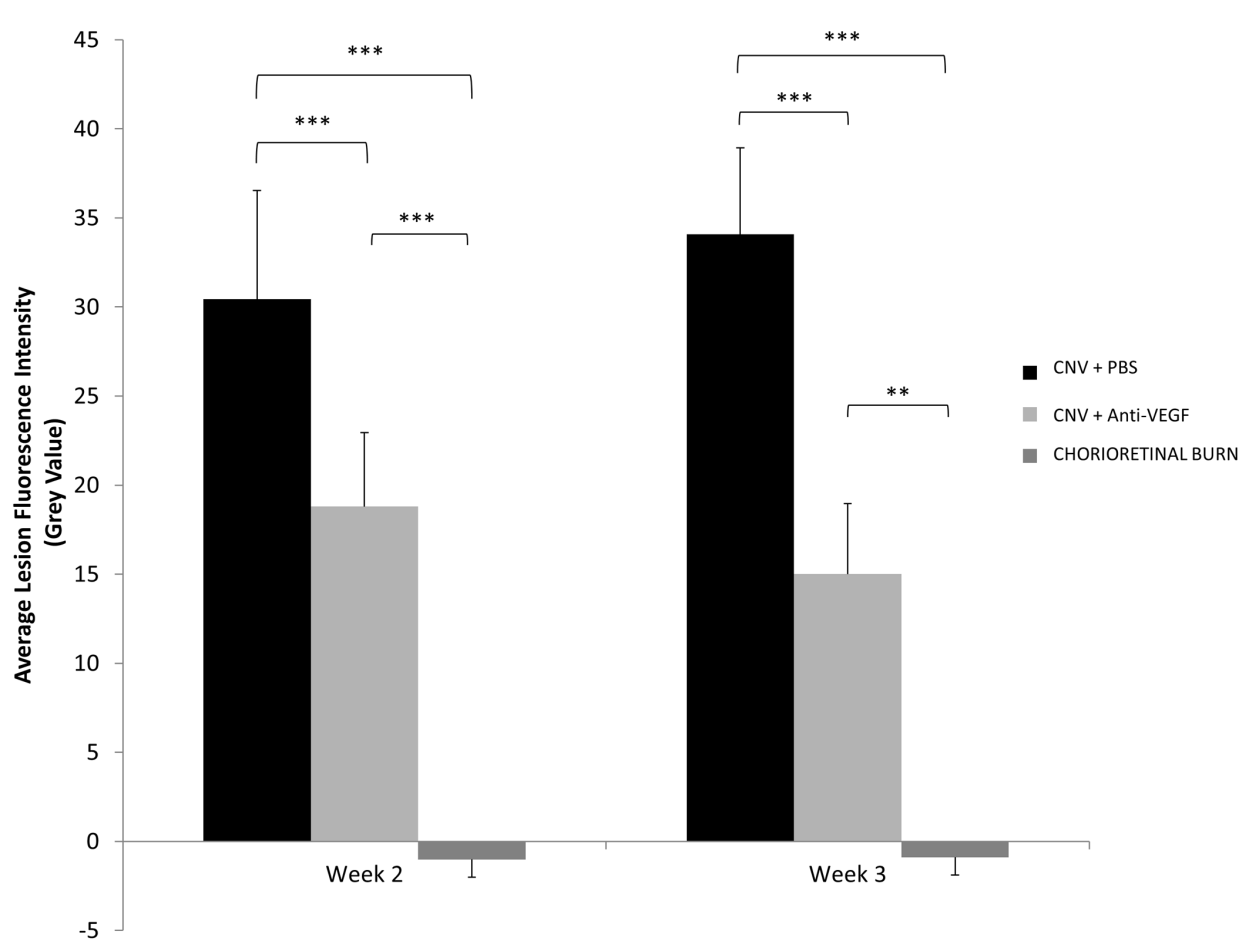
Fluorescein Angiogram CNV Net Fluorescence Analysis. Calculated net fluorescence above background of FFA images at 10.2 seconds post intravenous fluorescein injection of laser burns without CNV generation versus CNV lesions receiving anti-VEGF treatment and lesions receiving PBS. Values represent net average grey value ±SD (n = 16). ** Denotes statistically significant (*p*<0.01) difference in the calculated net fluorescence between treatment groups tested by two-tailed Student’s t-test. *** Denotes statistically significant (*p*<0.001) difference in the calculated net fluorescence between treatment groups tested by two-tailed Student’s t-test.

Net fluorescent intensity above local background was calculated for all lesions (Fig 6). All CR burns without generation of CNV exhibited classic hypofluorescent regions due to the lack of regular choroidal vessels, and the calculated negative net grey value remained approximately constant over time. Leakage of fluorescein from permeable CNV lesions result in areas of hyperfluorescence. Significantly (week 2, *p*<0.001; week 3, *p*<0.001) higher net fluorescence is observed from CNV lesions of eyes receiving PBS treatment than the avascular CR burn. Anti-VEGF treatment results in significantly (*p*<0.001) decreased fluorescent leakage from CNV lesions at week 2 and week 3 post treatment when compared with the PBS control. CNV lesions of PBS treated rats saw significant (*p* = 0.009) increase in net fluorescence between 2 and 3 weeks, all other treatment groups remained constant in net intensity.

### Area Corrected Lesion Fluorescence Intensity / Total Lesion Grey Value

**Fig 7:**
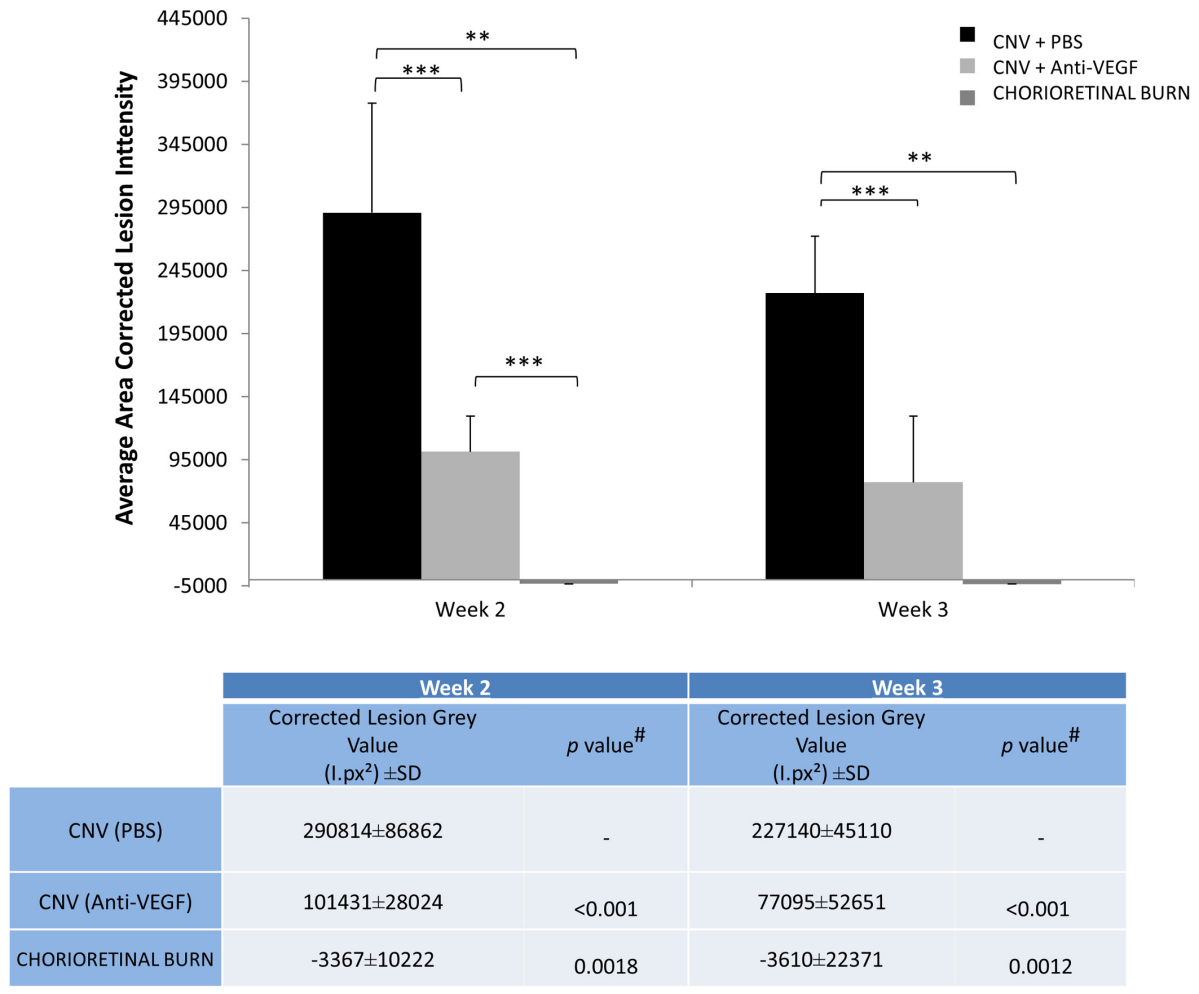
Area corrected lesion fluorescent intensity in fluorescein angiogram. Calculated average area corrected lesion fluorescent intensity of Laser Burn without CNV generation versus generated CNV with anti-VEGF treatment and PBS treatment. The ‘corrected lesion intensity’ of the hyperfluorescent region was calculated by multiplying the ‘net fluorescence intensity’ by the normalised calculated CNV area. Values represent net average grey value ±SD (n = 16). ** Denotes statistically significant (*p*<0.01) difference in the calculated total lesion grey value tested by two-tailed Student’s t-test. *** Denotes statistically significant (*p*<0.001) difference in the calculated total lesion grey value tested by two-tailed Student’s t-test. # *p* value determined by two-tailed Student’s t-test comparing respective experimental group with corrected lesion grey values from PBS treated rats.

The area corrected lesion intensity value provides a global assessment of CNV lesions as it incorporates measure of both net fluorescent intensity and normalised neovascular area (Fig 7) and allows discrimination of the severity of the lesions.

A significant (*p*<0.001) difference was observed between the anti-VEGF treated and PBS treated rats in the total lesion grey value of the lesions at week 2 and week 3. The avascular CR burn exhibited a corrected lesion grey value below that of background (attributed to regular choroidal vessels) with regular lesion size, while immediately post laser application the CNV lesions with PBS treatment exhibited very high corrected lesion intensity (Fig 7). Corrected lesion grey values of CNV lesions of anti-VEGF treated eyes exhibited an increase in variation with time. Variation in the calculated corrected lesion intensity is exaggerated in the arithmetic process of multiplication of the individual normalised lesion area and the net grey value.

## Discussion

Though the Micron III is a simple robust tool producing high-resolution fundus images and providing a platform for quantifiable CNV assessment, far superior to conventional grading schemes, no standard analytical method has been established so far. Of the available peer reviewed literature utilising the Micron III system, only one group attempted neovascularisation quantification associated with diabetic retinopathy [18], however the employed method using only grey-value threshold area analysis was overly simplistic, did not incorporate magnification correction and administration of fluorescein by intraperitoneal injection prohibited quantitative intensity measurements. To our knowledge, the present study is the first to establish *in vivo* CNV quantification methods using sequential FFA images from a rodent specific fundus camera and greatly reducing the need for large animal numbers while enabling longitudinal tracking of disease progression. Our method uses a combinational approach that incorporates a measure of 2-dimentional CNV area but also a measure of vessel integrity by way of intensity analysis of fluorescein exudate. Moreover, utilisation of common use software makes the method presented in this study accessible to all researchers.

### Image acquisition and its impact on analysis

Accurate quantification of CNV using this method is greatly dependent on clear fluorescein fundus images. As such, sources of error are related primarily to the operator at the acquisition stage rather than to the computer algorithms. With that in mind, care was taken to reduce inter animal variation, uphold image quality and reproducibility. In particular, corneal clarity, administration of fluorescein and alignment of the eye affect the fundus image significantly and consequently influence the results obtained.

Anesthetized rats with dilated pupils may develop cataract within few minutes, which may be related to corneal desiccation [28]. Cataracts will severely affect the ability take clear, focused fundus images, thus, we opted for a lower anaesthetic dose and immediately applied hypromellose coupling fluid or artificial tears to the cornea, following tropicamide application. Another factor affecting image quality is submaximal pupil dilation, resulting in a haze to the periphery of the image due to light scattering off the iris [29] obscuring peripheral lesions.

More significant light scattering will be introduced due to poor camera alignment with the pupillary axis. Off axis alignment of the eye with the lens system will also reduce the in-focus portion of the fundus and may slightly skew the image, this effect is more apparent in the periphery, to avoid this the camera and eye position was adjusted using standard white light colour fundus photography before administering the fluorescein ensuring correct alignment and focus on the optic nerve head plane. Animal respiration during the procedure may shift the fundus position slightly, in a 2 dimensional plane, however no significant impact on retinal focus is observed. During image acquisition, unavoidable pressure is exerted on the cornea by the lens system; the minute reduction in distance to the retina essentially imparts a magnification factor which is variable between animals and time points. This apparent magnification can be corrected by normalising the calculated CNV area against the size of a consistent anatomical feature such as the optic nerve head area, as described in the method. Furthermore using distance measurements between particular blood vessels as points of reference in stained retinal flatmounts applied to FFA images, in conjunction with routine choroidal flatmount preparation will allow better approximation of the CNV lesion size. However this technique is only applicable in retrospect and in our experience does not alter conclusions drawn from FFA.

The injection site, volume, concentration and delivery rate of the fluorescein influence not only the appearance time of CNV but also the intensity of the hyperfluorescent area. Intraperitoneal injection of fluorescein postpones arrival into the retinal vasculature and prolongs the peak florescence duration, at the expense of CNV definition, increased choroid background fluorescence and precludes intensity quantification due to altered perfusion rates (Fig 1). We observed better definition of hyperfluorescent areas using intravenous fluorescein via the lateral tail vein and intensity calculations were conducted using identical injection parameters between animals (fluorescein concentration, delivery rate, delivery site and volume). To reduce inter-animal CNV variation including age [26], gender [27] and general health, rats of a similar age and weight were selected and sexes were equally distributed between experimental groups. Fluorescein angiography was performed at the same time of day to avoid diurnal variation and approximately the same time after the anaesthetic dose delivered. Unlike clinical fundus cameras, where contrast is increased by nonlinear digital processing [30], Micron III uses linear processing at image capture, where digital values are recorded as linear function of light level [31]. This allows quantitative assessment of each pixel with regards to fluorescent intensity.

### Image analysis and accuracy

Due to poor lesion border definition observed at 1 week post laser application, even at peak fluorescence, CNV analysis by FFA is impractical. Studies interested in short term (<1 week) drug effects may find conventional choroidal flatmount or histopathological techniques more suitable. However, once the CNV has progressed sufficiently, quantification of fluorescein intensity and CNV area using our method can be performed. While the lasso tool in Photoshop is applicable for choroidal flatmount micrographs[13], we found the lesion border delineation in FFA images to be poor (data not shown), as such we opted for a manual technique under digital magnification using the freehand selection tool in ImageJ. Laser generated CNV lesions in PBS treated eyes remain approximately constant during the observation period, with respect to severity; confirming previously reported findings [32–34].

Based on our observations, any conclusion drawn on drug efficacy based on lesion area analysis alone from FFA images is not sufficient and often inappropriate, with smaller more severe and highly permeable lesions being misinterpreted.

Technical limitations of angiography must be taken into consideration, particularly as CNV area measurements rely on the persistence of fluorescein leaking from incompetent, newly formed permeable vessels. Misleading diffuse fluorescein leakage surrounding the CNV, may introduce error when outlining the maximal border of the CNV lesion from FFA images. One would expect FFA analysis to exhibit a larger standard deviation, than the conventional ex-vivo techniques, where blood vessel specific stains generate well defined lesions. However, strict delineation of lesion borders from high magnification micrographs of choroidal flat mounts, will include the elongated vascular budding at the lesion periphery, these projections will impact the overall lesion size calculation and contribute variation between lesions particularly in untreated or control animals where vascular budding is more apparent.

Once the CNV border was established, the average grey value was calculated. The contribution of normal retinal and choroidal capillaries to the CNV lesion fluorescence was subtracted. Given that the background fluorescence is mottled in appearance over the total retina, background fluorescence adjacent the CNV lesion was deemed a better representation of local microvasculature. However, net fluorescence values may misrepresent the observed CNV lesions as it is unable to distinguish between the severity of large highly permeable lesions from smaller equally permeable lesions. *In vivo* FFA analysis lends itself to the combined measure of area corrected fluorescent intensity, as it represents a value that establishes lesion severity by incorporating both a measure of CNV vessel integrity, as well as anatomical hyperfluorescent area. Indeed, traditional grading systems involve categorising lesions based on their severity, judged by CNV specialists, and not by select criteria such as size or intensity alone. Accordingly we multiplied the calculated lesion net fluorescence by the calculated CNV lesion area, normalised against the optic nerve head area, to establish a quantifiable value which incorporates both measurements.

The accuracy of the quantification method was tested by including an experimental group with an established method of CNV inhibition [21] and replicating the results using conventional choroidal flatmounting. As expected CNV area calculated using our FFA analysis technique indicated a significant difference in the size of CNV lesions of rats administered with anti-VEGF treatment than the PBS injected counterpart. We observed increased variability of CNV severity at week 3 in anti-VEGF treated rats; we postulate that the anti-VEGF antibody given immediately post laser and readministered at 7 days post laser, has been partially cleared from the vitreous of the animal [35] retracting angiogenic inhibition, or a delayed alternate angiogenic pathway had been promoted [36–38]. The net fluorescence of CNV lesions which receive anti-VEGF treatment (Fig 6) at week 2 and week 3 was significantly (p<0.001) less, indicating a clear reduction in vascular permeability associated with VEGF [39,40].

Our results shows that average ‘area corrected lesion intensity’ values obtained by FFA correlates with conventional choroidal flatmount techniques, demonstrating the power of *in vivo* imaging and the accuracy of the analysis method presented in this study; and provides additional valuable data regarding vessel permeability and lesion severity that is unobtainable in *ex vivo* assessment. *In vivo* imaging offers researchers a simple and commodious data source that can be used in conjunction with, or in lieu of conventional post mortem histopathology techniques and may improve our understanding of the dynamic nature of CNV.

### Future Technical Improvements

While we have presented an intentionally simplified analytical technique utilising common use software packages, incorporating measures to reduce technical errors and software function limitations, further revision of the methods we have employed and implementation of more sophisticated software would significantly improve CNV analysis. Our method, while somewhat automated, relies on the expertise of graders to outline the CNV lesion. Utilising advanced grey value thresholding may improve the system’s ability to delineate CNV area and integrated with the Micron III’s ability to record 30 FFA frames a second, potentially allows for a more sensitive, accurate and dynamic approach to CNV assessment. Adaptation of the algorithm presented by Serlin et al. [41] for human FFA image evaluation to include spatial assessment could provide real time information about CNV development and the efficacy of anti-angiogenic therapies and treatment methods.

## Conclusions

With recent developments in novel anti-angiogenic research, the need for a better standardised method of *in vivo* assessment of CNV has been highlighted. AMD research relies heavily on small rodent models to replicate the complex and dynamic processes involved in human retinopathology. The lack of implementation of *in vivo* quantification and evaluation of disease progression, has been a significant detriment to the field. The presented method was entirely developed using only open source or common use software packages and procedures used as simple as possible, with the intention to emphasise the accessibility and power of the technique to retinal research and promote the potential application to other high resolution fundus images. This method represents an important alternative to current conventional methods that preclude important long term *in vivo* tracking of neovascular disorders.

## References

[1] Resnikoff S, Pascolini D, Etya'ale D, Kocur I, Pararajasegaram R, Pokharel GP, et al (2004) Global data on visual impairment in the year 2002. Bull World Health Organ 82: 844–851. 15640920 PMC2623053

[2] Friedman DS, O'Colmain BJ, Munoz B, Tomany SC, McCarty C, de Jong PT, et al (2004) Prevalence of age-related macular degeneration in the United States. Arch Ophthalmol 122: 564–572. 15078675 10.1001/archopht.122.4.564

[3] Zarbin MA (1998) Age-related macular degeneration: review of pathogenesis. Eur J Ophthalmol 8: 199–206. 9891890 10.1177/112067219800800401

[4] Grossniklaus HE, Kang SJ, Berglin L (2010) Animal models of choroidal and retinal neovascularization. Prog Retin Eye Res 29: 500–519. 10.1016/j.preteyeres.2010.05.003 20488255 PMC2962694

[5] Dobi ET, Puliafito CA, Destro M (1989) A new model of experimental choroidal neovascularization in the rat. Arch Ophthalmol 107: 264–269. 2464985 10.1001/archopht.1989.01070010270035

[6] Frank RN, Das A, Weber ML (1989) A model of subretinal neovascularization in the pigmented rat. Curr Eye Res 8: 239–247. 2468453 10.3109/02713688908997565

[7] Tobe T, Ortega S, Luna JD, Ozaki H, Okamoto N, Derevjanik NL, et al (1998) Targeted disruption of the FGF2 gene does not prevent choroidal neovascularization in a murine model. Am J Pathol 153: 1641–1646. 9811357 10.1016/S0002-9440(10)65753-7 PMC1853405

[8] Seo MS, Kwak N, Ozaki H, Yamada H, Okamoto N, Yamada E, et al (1999) Dramatic inhibition of retinal and choroidal neovascularization by oral administration of a kinase inhibitor. Am J Pathol 154: 1743–1753. 10362799 10.1016/S0002-9440(10)65430-2 PMC1866636

[9] Ryan SJ (1979) The development of an experimental model of subretinal neovascularization in disciform macular degeneration. Trans Am Ophthalmol Soc 77: 707–745. 94717 PMC1311723

[10] Campos M, Amaral J, Becerra SP, Fariss RN (2006) A novel imaging technique for experimental choroidal neovascularization. Invest Ophthalmol Vis Sci 47: 5163–5170. 17122098 10.1167/iovs.06-0156

[11] Campa C, Kasman I, Ye W, Lee WP, Fuh G, Ferrara N (2008) Effects of an anti-VEGF-A monoclonal antibody on laser-induced choroidal neovascularization in mice: optimizing methods to quantify vascular changes. Invest Ophthalmol Vis Sci 49: 1178–1183. 10.1167/iovs.07-1194 18326747

[12] Lambert V, Lecomte J, Hansen S, Blacher S, Gonzalez ML, Struman I, et al (2013) Laser-induced choroidal neovascularization model to study age-related macular degeneration in mice. Nat Protoc 8: 2197–2211. 10.1038/nprot.2013.135 24136346

[13] Toma HS, Barnett JM, Penn JS, Kim SJ (2010) Improved assessment of laser-induced choroidal neovascularization. Microvasc Res 80: 295–302. 10.1016/j.mvr.2010.05.011 20553963 PMC3390000

[14] Zhou X, Bedggood P, Metha A (2012) Limitations to adaptive optics image quality in rodent eyes. Biomed Opt Express 3: 1811–1824. 10.1364/BOE.3.001811 22876346 PMC3409701

[15] Geng Y, Greenberg KP, Wolfe R, Gray DC, Hunter JJ, Dubra A, et al (2009) In vivo imaging of microscopic structures in the rat retina. Invest Ophthalmol Vis Sci 50: 5872–5879. 10.1167/iovs.09-3675 19578019 PMC2873188

[16] Criswell MH, Hu WZ, Steffens TJ, Li R, Margaron P (2008) Comparing pegaptanib and triamcinolone efficacy in the rat choroidal neovascularization model. Arch Ophthalmol 126: 946–952. 10.1001/archopht.126.7.946 18625941

[17] Funakoshi T, Birsner AE, D'Amato RJ (2006) Antiangiogenic effect of oral 2-methoxyestradiol on choroidal neovascularization in mice. Exp Eye Res 83: 1102–1107. 16828472 10.1016/j.exer.2006.05.016

[18] Qu Y, Zhang S, Xu X, Wang H, Li J, Zhou F, et al (2009) Octreotide inhibits choroidal neovascularization in rats. Ophthalmic Res 42: 36–42. 10.1159/000219683 19478539

[19] Nishiwaki H, Zeimer R, Goldberg MF, D'Anna SA, Vinores SA, Grebe R (2002) Laser targeted photo-occlusion of rat choroidal neovascularization without collateral damage. Photochemistry and Photobiology 75: 149–158. 11883603 10.1562/0031-8655(2002)075<0149:ltpoor>2.0.co;2

[20] Lu F, Adelman RA (2009) Are intravitreal bevacizumab and ranibizumab effective in a rat model of choroidal neovascularization? Graefes Arch Clin Exp Ophthalmol 247: 171–177. 10.1007/s00417-008-0936-y 18781316

[21] Wang W, Wang F, Lu F, Xu S, Hu W, Huang J, et al (2011) The antiangiogenic effects of integrin alpha5beta1 inhibitor (ATN-161) in vitro and in vivo. Invest Ophthalmol Vis Sci 52: 7213–7220. 10.1167/iovs.10-7097 21813636

[22] Wang JL, Liu YL, Li Y, Dai WB, Guo ZM, Wang ZH, et al (2012) EphA2 targeted doxorubicin stealth liposomes as a therapy system for choroidal neovascularization in rats. Invest Ophthalmol Vis Sci 53: 7348–7357. 10.1167/iovs.12-9955 22977140

[23] Han Z, Guo J, Conley SM, Naash MI (2013) Retinal angiogenesis in the Ins2(Akita) mouse model of diabetic retinopathy. Invest Ophthalmol Vis Sci 54: 574–584. 10.1167/iovs.12-10959 23221078 PMC3558298

[24] Huang H, Parlier R, Shen JK, Lutty GA, Vinores SA (2013) VEGF receptor blockade markedly reduces retinal microglia/macrophage infiltration into laser-induced CNV. PLoS One 8: e71808 10.1371/journal.pone.0071808 23977149 PMC3748119

[25] Guaiquil VH, Hewing NJ, Chiang MF, Rosenblatt MI, Chan RV, Blobel CP (2013) A murine model for retinopathy of prematurity identifies endothelial cell proliferation as a potential mechanism for plus disease. Invest Ophthalmol Vis Sci 54: 5294–5302. 10.1167/iovs.12-11492 23833070 PMC3738219

[26] Espinosa-Heidmann DG, Suner I, Hernandez EP, Frazier WD, Csaky KG, Cousins SW (2002) Age as an independent risk factor for severity of experimental choroidal neovascularization. Investigative Ophthalmology & Visual Science 43: 1567–1573. 11980875

[27] Tanemura M, Miyamoto N, Mandai M, Kashii S, Honda Y (2003) Estrogen may enhance laser-induced CNV formation by enhancing VEGFR2 gene expression. Investigative Ophthalmology & Visual Science 44: U109–U109.

[28] Calderone L, Grimes P, Shalev M (1986) Acute reversible cataract induced by xylazine and by ketamine-xylazine anesthesia in rats and mice. Exp Eye Res 42: 331–337. 3754819 10.1016/0014-4835(86)90026-6

[29] Kim HM, Lowery JC, Kurtz R (2007) Accuracy of digital images for assessing diabetic retinopathy. J Diabetes Sci Technol 1: 531–539. 19885116 10.1177/193229680700100411 PMC2769632

[30] Schalenbourg A, Zografos L (2013) Pitfalls in colour photography of choroidal tumours. Eye (Lond) 27: 224–229. 10.1038/eye.2012.267 23238442 PMC3574260

[31] Hardin W (2010) Revealing Eye. Vision System Designs 15.

[32] Edelman JL, Castro MR (2000) Quantitative image analysis of laser-induced choroidal neovascularization in rat. Exp Eye Res 71: 523–533. 11040088 10.1006/exer.2000.0907

[33] Zhao SH, He SZ (2003) Study on the experimental model of krypton laser-induced choroidal neovascularization in the rats. Zhonghua Yan Ke Za Zhi 39: 298–302. 12892608

[34] Cui J, Liu Y, Zhang J, Yan H (2014) An experimental study on choroidal neovascularization induced by krypton laser in rat model. Photomed Laser Surg 32: 30–36. 10.1089/pho.2013.3588 24328846

[35] Lin YS, Nguyen C, Mendoza JL, Escandon E, Fei D, Meng YG, et al (1999) Preclinical pharmacokinetics, interspecies scaling, and tissue distribution of a humanized monoclonal antibody against vascular endothelial growth factor. J Pharmacol Exp Ther 288: 371–378. 9862791

[36] Hu Y, Chen Y, Lin M, Lee K, Mott RA, Ma JX (2013) Pathogenic role of the Wnt signaling pathway activation in laser-induced choroidal neovascularization. Invest Ophthalmol Vis Sci 54: 141–154. 10.1167/iovs.12-10281 23211829 PMC3544418

[37] Zhu J, Wang YS, Zhang J, Zhao W, Yang XM, Li X, et al (2009) Focal adhesion kinase signaling pathway participates in the formation of choroidal neovascularization and regulates the proliferation and migration of choroidal microvascular endothelial cells by acting through HIF-1 and VEGF expression in RPE cells. Exp Eye Res 88: 910–918. 10.1016/j.exer.2008.11.034 19111720

[38] Xie B, Shen J, Dong A, Rashid A, Stoller G, Campochiaro PA (2009) Blockade of sphingosine-1-phosphate reduces macrophage influx and retinal and choroidal neovascularization. J Cell Physiol 218: 192–198. 10.1002/jcp.21588 18781584 PMC2905312

[39] Leung DW, Cachianes G, Kuang WJ, Goeddel DV, Ferrara N (1989) Vascular Endothelial Growth-Factor Is a Secreted Angiogenic Mitogen. Science 246: 1306–1309. 2479986 10.1126/science.2479986

[40] Aiello LP, Bursell SE, Clermont A, Duh E, Ishii H, Takagi C, et al (1997) Vascular endothelial growth factor-induced retinal permeability is mediated by protein kinase C in vivo and suppressed by an orally effective beta-isoform-selective inhibitor. Diabetes 46: 1473–1480. 9287049 10.2337/diab.46.9.1473

[41] Serlin Y, Tal G, Chassidim Y, Parmet Y, Tomkins O, Knyazer B, et al (2013) Novel fluorescein angiography-based computer-aided algorithm for assessment of retinal vessel permeability. PLoS One 8: e61599 10.1371/journal.pone.0061599 23626701 PMC3634003

